# PEGylation of lipophilic SN38 prodrug with DSPE-mPEG_2000_ versus cremophor EL: comparative study for intravenous chemotherapy

**DOI:** 10.1080/10717544.2019.1587045

**Published:** 2019-03-26

**Authors:** Jun Zeng, Chen Li, Xing Duan, Fuyue Liu, Anqin Li, Chunhan Luo, Li Jia, Yifang Gan, Lu Yan, Yaxin Zheng

**Affiliations:** a Division of Pulmonary and Critical Care Medicine, The First Affiliated Hospital of Chengdu Medical College, Chengdu Medical College, Chengdu, China;; b School of Pharmacy, Key Laboratory of Sichuan Province for Specific Structure of Small Molecule Drugs, Chengdu Medical College, Chengdu, China

**Keywords:** SN38, lipophilic prodrug, nanomedicine, Cremophor EL, PEGylation

## Abstract

The lipophilic prodrug of hydrophobic drugs with well-designed molecular structures can form stable pure prodrug nanoparticles (NPs), but rapid NPs aggregation in plasma greatly restricted their direct use for intravenous chemotherapy. To address this, DSPE-mPEG_2000_ and Cremophor EL are two of the most widely used lipophilic PEG derivatives to enhance their colloidal stability in plasma. However, their drug delivery performances have never been comparatively studied. Here, a redox-responsive lipophilic prodrug of SN38 was chosen as the model drug for such comparative investigations. We found that Cremophor EL/NPs having a small diameter (∼15 nm) and poor kinetic stability displayed an enhanced cell internalization, higher cytotoxicity and prolonged circulation time as compared with DSPE-mPEG_2000_/NPs. Most importantly, these superiorities further resulted in a much more potent antitumor activity in CT26 colorectal cancer xenograft, but the increased loss of body weight was also noted. These results suggested that Cremophor EL could be more advantageous than DSPE-mPEG_2000_ in terms of the improvement of antitumor activity, but the enhanced toxicity warranted further attention in the future study.

## 1. Introduction

Lipids have been widely conjugated to various hydrophobic drugs to obtain the so-called dually hydrophobic prodrugs (DHPs). By enhancing their loading capacity in the lipid-based nanocarriers, DHPs have been regarded as a feasible strategy to address the solubility problem of hydrophobic chemotherapeutic agents (Lundberg, 2011; Bala et al., 2016; Ren et al., 2016). Recently, DHPs containing particular chemical elements (e.g. unsaturated fatty acid, disulfide bond and carbonate esters) are found to be able to self-assemble into pure prodrug nanoaggregates in water, despite of the absence of a typical amphiphilic structure (Dosio et al., 2010; Wang Y et al., 2014; Wang H et al., 2015; Li et al., 2018). Advantages of nanoaggregates formed by small-molecule DHPs include precisely defined chemical structures, high loading capacity (Zhang et al., 2016), good thermodynamic stability (Li et al., 2018), enhanced tumor targeting and reduced toxicity (Bradley et al., 2001a,b). Therefore, DHP strategy has recently received considerable attention in the field of nanomedicine (Wang Y et al., 2017; Zhang et al., 2017a,b; Sun et al., 2018; Zhong et al., 2018).

Despite so many benefits, DHP nanoaggregates usually form the extensive aggregation in the phosphate buffer saline (PBS) or plasma (Zheng et al., 2019), which further results in the quick clearance of nanoaggregates from the bloodstream postintravenous injections (Wang Y et al., 2014; Luo et al., 2016). To address this, various lipophilic PEG derivatives have been used to enhance the colloidal stability of DHP nanoaggregates in physiologically relevant media. In an effort to develop the stable DHP nanoaggregates, DSPE-mPEG_2000_ is the most efficient option due to its good biocompatibility and long history of clinical usage. Modifying DHP nanoaggregates with only 10 ∼ 15% (w/w) DSPE-mPEG_2000_ can result in a significant improvement in the blood circulation times, without the compromising loading capacity (Wang Y et al., 2014; Li et al., 2018).

However, DSPE-mPEG_2000_ has not been extensively utilized for the formulation of DHPs in the clinical trials. For example, the most well-known DHP in the clinical trials, the conjugate of docosahexaenoic acid and paclitaxel (DHA-PTX), was formulated in the 10% Cremophor EL (Wolff et al., 2003). The resulting DHA-PTX injection not only shows a remarkably 376 times higher plasma AUC than that of Taxol, but also displays around 57-fold higher tumor accumulation (Bradley et al., 2001a; Wolff et al., 2003). Such superior pharmacokinetics and biodistributions further result in the impressively enhanced antitumor activity (nearly 100% complete regressions) as compared with Taxol at the equitoxic dose in the tumor-bearing mice (Bradley et al., 2001a). The most recent clinical outcomes of DHA-PTX at phase-III trial on patients with metastatic malignant melanoma fell short of expectations. However, this is supposedly attributed to the extremely slow drug release from the ester-linked DHA-PTX, rather than to the use of Cremophor EL (Bedikian et al., 2011). Recently, it has been demonstrated that DHP containing a redox-responsive linker (i.e. disulfide bond) displayed a significantly enhanced antitumor efficacy than that of ester-linked DHP at the same dose. This was mainly ascribed to the enhanced drug release responding to the intracellular glutathione (GSH) overproduced in tumor cells (Luo et al., 2016a,b). Therefore, there is a need to explore the deliver performance of such redox-responsive DHPs formulated in Cremophor EL.

Although both DSPE-mPEG_2000_ and Cremophor EL have been extensively used for the PEGylation of DHPs for intravenous chemotherapy (Bradley et al., 2001a,b; Ke et al., 2010; Wang Y et al., 2014; Li et al., 2018), their comparative investigation has never been performed, especially for a redox-sensitive DHP. We have recently reported on the self-assembling nanoparticles (NPs) composed of the redox-sensitive DHP of 7-ethyl-10-hydroxyl-camptothecin (SN38). The resulting NPs were further PEGylated with DSPE-mPEG_2000_ to inhibit aggregation in plasma, and *in vivo* antitumor efficacy was demonstrated in the CT26 colorectal cancer xenograft (Zheng et al., 2019). In this study, the lipophilic SN38 prodrug was also formulated in Cremophor EL, and their characterizations and drug delivery performances (i.e. cellular uptake, cytotoxicity, pharmacokinetics and *in vivo* antitumor efficacy) were compared with DSPE-mPEG_2000_ modified NPs. The results represented here would highlight the great different effects of two widely-used PEGylation strategies on the drug delivery performance and chemotherapeutic efficacy of DHPs.

## 2. Materials and methods

### 2.1. Materials and animals

The SN38, Curcumin (Cur), LysoTracker, AannexinV-FITC apoptosis detection kit were purchased from MEILUN Biology Technology Co., LTD. (Dalian, China). The DSPE-mPEG_2000_ was purchased from Lipoid GmbH (Ludwigshafen, Germany). Cremophor EL was obtained from BASF (Ludwigshafen, Germany). Lipophilic Cur prodrug was synthesized according to previous report (Li et al., 2018). All solvents used in this study were analytical grade. Male BALB/c mice (6–8 weeks old) and Sprague–Dawley rats (200–250 g) were purchased from the Laboratory Animal Center of Chengdu Medical college (Chengdu, China). All studies involving mice were approved by the institute’s animal care and use committee.

### 2.2. Preparation of DSPE-mPEG_2000_/NPs and cremophor EL/NPs of lipophilic SN38 prodrug

The redox-responsive lipophilic SN38 prodrug was synthesized according to our previous report, the structure was confirmed by ^1^H-NMR (Zheng et al., 2019). SN38 prodrug-loaded DSPE-mPEG_2000_/NPs were prepared according to the nanoprecipitation method. Briefly, ethanol solution of lipophilic SN38 prodrug (10 mg/ml) and DSPE-mPEG_2000_ (1 mg/ml) was dispersed dropwise into distilled water under vigorous agitation. The ethanol was then removed under reduced pressure at 45 °C. Cremophor EL/NPs were prepared by diluting the lipophilic SN38 prodrug solution (10 mg/ml) in ethanol/Cremophor EL (5/1, v/v) into distilled water to reach a desired concentration.

To compare the self-assembling behavior of DSPE-mPEG_2000_/NPs and Cremophor EL/NPs, the effects of added amount of DSPE-mPEG_2000_ or Cremophor EL on their particle sizes and zeta potentials were comparatively investigated. The ratios of lipophilic SN38 prodrug to DSPE-mPEG_2000_ or Cremophor EL were set at 1:17, 1:1.7 and 1:0.1 (w/w), respectively. These ratios were determined according to their frequently-used doses in the previous reports (Bradley et al., 2001a; Wolff et al., 2003; Wang Y et al., 2014; Li et al., 2018)

### 2.3. Characterization of NPs

The size, size distribution and zeta potential of NPs were measured using Zetasizer (Nano-ZS90, Malvern, England) at 25 °C. Transmission electron microscopy (TEM, JEM-1200EX, Japan) was utilized to observe the NP morphology, samples were stained with 2% uranyl acetate. Storage stability of DSPE-mPEG_2000_/NPs and Cremophor EL/NPs were investigated by measuring their particle sizes in water stored at 4 °C for 4 weeks, three samples of each version of NPs were used for the evaluation. Aggregation states of lipophilic SN38 prodrug in DSPE-mPEG_2000_/NPs and Cremophor EL/NPs were investigated using UV-vis spectra and fluorescence emission spectra (λ_ex_ = 362 nm and λ_em_ = 380–600 nm) at the SN38 equivalent concentration of 10 µg/ml, respectively.

Kinetic stabilities of DSPE-mPEG_2000_/NPs and Cremophor EL/NPs were evaluated using a fluorescence resonance energy transfer (FRET) technique. Briefly, a lipophilic prodrug of Cur was chosen as the FRET acceptor, with the lipophilic SN38 prodrug itself as FRET donor. The SN38 prodrug-loaded NPs were mixed with NPs containing Cur prodrug in a 96-well plate (mole/mole, 1/1). The mixture was then incubated at 37.5 °C, SN38 fluorescence was determined by a fluorescence spectrometer at the given time interval (λ_ex_= 362 nm and λ_em_ = 426 nm). The SN38 fluorescence would be quenched due to the exchange of Cur prodrug or SN38 prodrug between NPs, which could be used to evaluate their kinetic stability (Owen et al., 2012).

In vitro release of SN38 from DSPE-mPEG_2000_/NPs and Cremophor EL/NPs in the culture medium were comparatively studied. Briefly, DSPE-mPEG_2000_/NPs and Cremophor EL/NPs were supplemented with 1 ml of fresh culture medium at the final SN38 equivalent concentration of 10 µg/ml. The samples were then incubated in a water bath at 37 °C. At the given time interval, 100 μl of sample was withdrawn and extracted with 300 μl methanol (containing 1% acetic acid) for HPLC analysis.

### 2.4. Cellular uptake

Colorectal cancer cells (CT26) were purchased from American Type Culture Collection (Rockville, MD, USA). CT26 cells were maintained in RPMI 1640 containing 10% FBS, penicillin (100 units/ml) and streptomycin (100 μg/ml) in a humidified atmosphere of 5% CO_2_ at 37 °C. The cells were exposed to SN38 prodrug-loaded DSPE-mPEG_2000_/NPs and Cremophor EL/NPs, and incubated at an equivalent SN38 concentration of 10 μg/ml for 2 h at 37 °C, respectively. Then, cells were washed three times with cold PBS and fixed with 4% paraformaldehyde for 30 min. The cells were further incubated with LysoTracker at a concentration of 50 nM for 1.5 h to label lysosomes. The images of cells were observed using a confocal laser scanning microscopy (Nikon, USA).

To quantitatively determine the cellular uptake of SN38 prodrug and SN38 released in the cytoplasm, CT26 cells were exposed to DSPE-mPEG_2000_/NPs and Cremophor EL/NPs at SN38 equivalent doses of 5 μg/ml for 4 h at 37 °C. The medium was then removed and cells were washed with cold PBS three times. 300 μl methanol (containing 1% acetic acid) was added to each well. The cell lysate was centrifuged at 10,000 rpm for 10 min, and 20 μl of the supernatant was injected to HPLC system for quantification.

### 2.5. Cytotoxicity assay

The cell viability was assessed by MTT assay. Briefly, CT26 cells were seeded in a 96-well plate at a density of approximate 5000 cells per well. After 24 h of growth, the medium was exchanged for the medium that contained DSPE-mPEG_2000_/NPs and Cremophor EL/NPs at various concentrations. The cells were further incubated for 48 h, and these without any treatment were utilized as control.

### 2.6. Cell apoptosis analysis

CT26 cells in 12-well plate (5 × 10^4^ cell/well) were incubated with DSPE-mPEG_2000_/NPs and Cremophor EL/NPs at the SN38 equivalent doses of 5 μg/ml for 48 h at 37 °C, then collected consecutively by trypsinization and centrifugation and washed with cold PBS for several times. The cells were stained with annexin V-FITC and propidium iodide (PI) using the AannexinV-FITC apoptosis detection kit. The apoptosis analysis was performed on a flow cytometer (NovoCyte 3130, ACEA, USA).

### 2.7. Pharmcokinetics

Sprague–Dawley rats (200–250 g) were used for the pharmacokinetics studies. Male Sprague–Dawley rats (4 rats/per group) were assigned to one of two groups, which received an intravenous bolus of DSPE-mPEG_2000_/NPs and Cremophor EL/NPs at an equivalent SN38 dose of 5 mg/kg. At the indicated time points, about 0.4 ml blood samples were taken and centrifuged to obtain the plasma sample. The concentration of prodrugs and free SN38 in plasma was analyzed by a validated HPLC method.

### 2.8. *In vivo* antitumor efficacy

A subcutaneous model of colorectal cancer was established by subcutaneously injecting CT26 cells (2 × 10^6^ cells per 100 μl) into the right axillary flank region of Male BALB/C mice. Five days later, the mice were randomly assigned to 3 groups (*n* = 7) and intravenously injected with multiple injections (every 2 days × 5) of saline, DSPE-mPEG_2000_/NPs and Cremophor EL/NPs at equivalent SN38 dose of 10 mg/kg. All the mice were sacrificed two days post the final injection, and all tumors were harvested and weighed.

## 3. Results and discussion

### 3.1. Preparation and characterization

**Figure 1.**
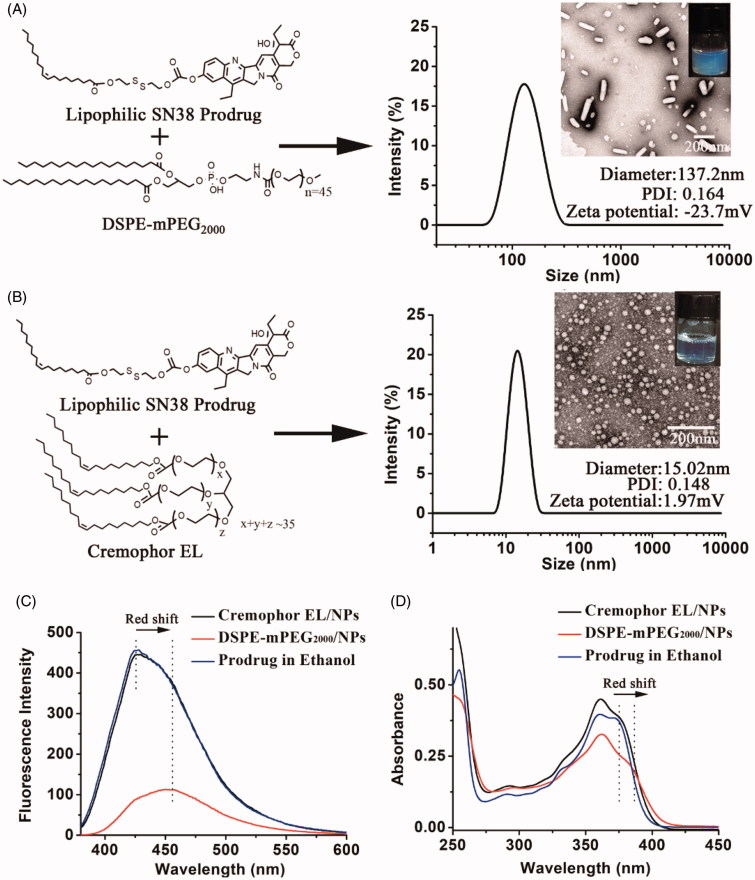
Preparation and characterization (TEM image, zeta potential, size distribution and PDI) of DSPE-mPEG_2000_/NPs (A) and Cremophor EL/NPs (B). Fluorescence emission spectra (C) and UV-vis spectra (D) of the DSPE-mPEG_2000_/NPs, Cremophor EL/NPs and ethanol solution of SN38 prodrug (monomeric species) at the SN38 equivalent concentration of 10 µg/ml.

DSPE-mPEG_2000_/NPs were prepared by dispersing the ethanol solution of SN38 prodrug and DSPE-mPEG_2000_ into distilled water under vigorous agitation. As shown in Figure 1(A), DSPE-mPEG_2000_/NPs showed a rod-like morphology with an average hydrodynamic diameter of 137.2 nm and a zeta potential of −23.7 mV. However, TEM also revealed many small sphere-shaped NPs, which might be the micelles formed by DSPE-mPEG_2000_ (Kastantin et al., 2010). Cremophor EL/NPs were prepared by diluting the SN38 prodrug solution (ethanol/Cremophor EL, 5/1, v/v) into the distilled water. The quantity of Cremophor EL used for the preparation of Cremophor EL/NPs was only around 20% of Taxol, which was according to the preparation of DHA-PTX injection in the clinical trials (Bradley et al., 2001a,b; Bedikian et al., 2011). As shown in Figure 1(B), Cremophor EL/NPs displayed completely different properties from those of DSPE-mPEG_2000_/NPs, such as the homogeneous sphere morphologies with sharply decreased particle size (∼15 nm) and nearly neutral surface (zeta potential, 1.97 mV). Cremophor EL/NPs with such a small particle size were supposed to show an enhanced tumor penetration than that of DSPE-mPEG_2000_/NPs, as the small NPs have been demonstrated to penetrate the tumor much more readily than those with a larger size (Cabral et al., 2011; Wang Y et al., 2015).

The aggregation state of lipophilic SN38 prodrug in DSPE-mPEG_2000_/NPs and Cremophor EL/NPs was investigated using UV-vis spectra and fluorescence emission spectra. As shown in Figure 1(C), the emission spectrum of DSPE-mPEG_2000_/NPs displayed much lower fluorescence intensity than that of SN38 prodrug in ethanol, indicating an aggregation-caused fluorescence quenching. Similar results have been observed in the previous reports (Zhang et al., 2016; Hou et al., 2018). Furthermore, both UV-vis spectrum and emission spectrum of DSPE-mPEG_2000_/NPs showed an apparent bathochromic peak, whereas spectra of Cremophor EL/NPs were just similar to that of ethanol solution of SN38 prodrug (existing in monomeric species) (Figure 1(C–D)). These results suggested that the lipophilic SN38 prodrugs in Cremophor EL/NPs were much less aggregated than that in DSPE-mPEG_2000_/NPs.

The remarkably different size, zeta potential, morphology and aggregation state might arise from their different assembling mechanism. Note that the quantity of DSPE-mPEG_2000_ used for the preparation of DSPE-mPEG_2000_/NPs was only 10% of that of lipophilic SN38 prodrug (w/w). Therefore, DSPE-mPEG_2000_ was expected to insert merely onto the surface of nanoaggregates consisting of SN38 prodrugs. Due to presence of polar carbonyl with a high negative charge density, the pure NPs of lipophilic SN38 prodrugs had a high negative zeta potential (−30.3 mV) (Zheng et al., 2019). Therefore, the anchored DSPE-mPEG_2000_ (itself containing one negative charge) could only shield the part negative charges by moving the hydrodynamic shear plane from the charged surface to the edge of the PEG coating. For Cremophor EL/NPs, the quantity of Cremophor EL was nearly 17.5 times that of SN38 prodrug, and the similar particle size was observed between the SN38 prodrug-loaded and blank Cremophor EL/NPs (13.3 nm). Therefore, lipophilic SN38 prodrugs were speculated to be encapsulated in the hydrocarbon palisade region of Cremophor EL micelles, which could be supported by their less aggregated stage and nearly neutral zeta potential similar to that of blank Cremophor EL micelles (−2.8 mV). For this reason, Cremophor EL/NPs should be regarded as micelles in nature. It is worth mentioning that the quantities of Cremophor EL and DSPE-mPEG_2000_ were based on their frequently-used doses in the previous reports (Bradley et al., 2001a; Wolff et al., 2003; Wang Y et al., 2014; Li et al., 2018).

To better understand the self-assembly of DSPE-mPEG_2000_/NPs and Cremophor EL/NPs, effects of the amount of stabilizer (lipophilic PEG derivatives) on their particle sizes and zeta potentials were comparatively investigated. As shown in Table 1, both particle sizes and zeta potentials of Cremophor EL/NPs were gradually decreased with the increasing of prodrug/stabilizer ratios, whereas DSPE-mPEG_2000_/NPs showed relatively larger particles (>100 nm) with high negative zeta potentials, irrespective of prodrug/stabilizer ratios. This result indicated that lipophilic SN38 prodrug could be readily encapsulated into the Cremophor EL micelles rather than DSPE-mPEG_2000_ micelles. This result was in good agreement with the previous report (Zhong et al., 2016), in which the enhanced amount of DSPE-mPEG_2000_ failed to decrease the particle sizes of linoleic acid-paclitaxel conjugate-loaded DSPE-mPEG_2000_/NPs to the value similar to that of the blank DSPE-mPEG_2000_ micelles.

**Table 1.** Effects of added amount of lipophilic PEG derivatives on particles size (zeta potential) of DSPE-mPEG_2000_/NPs and Cremophor EL/NPs.

Prodrug/Stabilizer (w/w)	Cremophor EL/NPs			DSPE-mPEG_2000_/NPs		
	Size (nm)	PDI	Zeta potential (mV)	Size (nm)	PDI	Zeta potential (mV)
1:17.5	15.02	0.148	1.97	178.5	0.245	−34.1
1:1.75	80.46	0.205	−7.61	104.2	0.274	−23.5
1:0.1	121.2	0.143	−15.6	137.2	0.164	−23.7

### 3.2. Storage stability and kinetic stability

**Figure 2.**
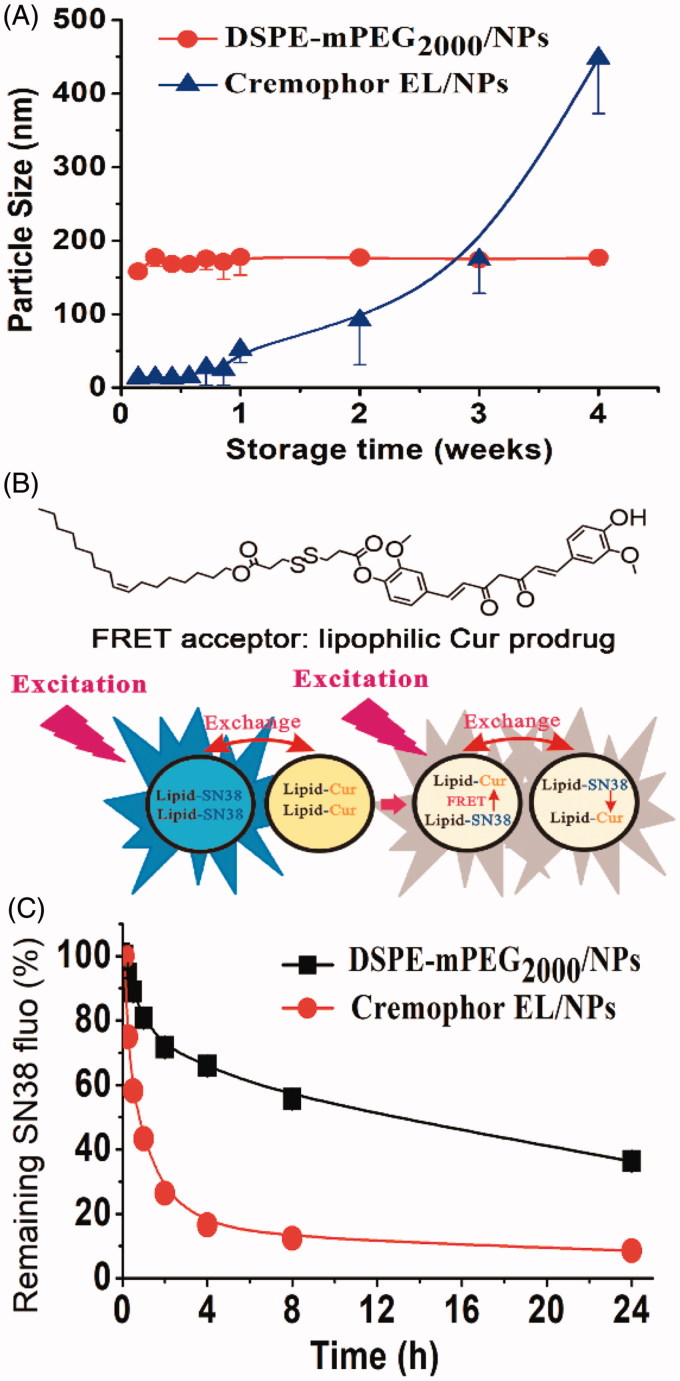
Change of particle size of DSPE-mPEG_2000_/NPs and Cremophor EL/NPs in water stored at 4 °C for 4 weeks, [means ± SD, *n* = 3] (A). Schematic illustration of the mechanism to detect prodrug exchange between NPs using a FRET technique, the lipophilic prodrugs of SN38 and Cur were chosen as the FRET donor and acceptor, respectively (B). Kinetic change of fluorescence intensity of SN38 when SN38 prodrug-loaded NPs were mixed with that containing Cur prodrug (C).

Storage stability of Cremophor EL/NPs and DSPE-mPEG_2000_/NPs was evaluated by analyzing their particle size during storage at 4 °C for 4 weeks. As shown in Figure 2(A), DSPE-mPEG_2000_/NPs showed no significant change in the size during storage, whereas Cremophor EL/NPs exhibited a significantly increased size. This result suggested that Cremophor EL/NPs displayed a much poorer storage stability than that of DSPE-mPEG_2000_/NPs.

Such remarkable difference in storage stability was believed to arise from their distinct kinetic stability, which was directly related to the exchange of the SN38 prodrug molecules between NPs (Owen et al., 2012). To confirm this, we used a FRET technique to investigate the exchange of SN38 prodrug between NPs. Schematic illustration of the mechanism to detect prodrug exchange was shown in Figure 2(B). A lipophilic prodrug of Cur was chosen as the FRET acceptor (Li et al., 2018), with the lipophilic SN38 prodrug itself as FRET donor. When SN38 prodrug-loaded NPs were mixed with NPs containing Cur prodrug, the exchange of Cur or SN38 prodrug between NPs would result in the quenching of SN38 fluorescence. By capturing the kinetic change of SN38 fluorescence, the exchange rate of prodrugs could be monitored in a real-time manner.

We next mixed SN38 prodrug-loaded NPs with that containing Cur prodrug at the SN38/Cur ratio of 1/1 (mole/mole). The NPs mixtures were then incubated at 37.5 °C for 24 h, and their kinetic changes of SN38 fluorescence at 426 nm were recorded with the excitation at 362 nm. As shown in Figure 2(C), Cremophor EL/NPs displayed a more rapid decrease of SN38 fluorescence than that of DSPE-mPEG_2000_/NPs, indicating a faster prodrug exchange and poorer kinetic stability. The poor kinetic stability of Cremophor EL/NPs might arise from the presence of the large amount of Cremophor EL, which facilitated the prodrug exchange through the merging and splitting of Cremophor EL micelles (Owen et al., 2012).

### 3.3. Cellular uptake and intracellular localization

**Figure 3.**
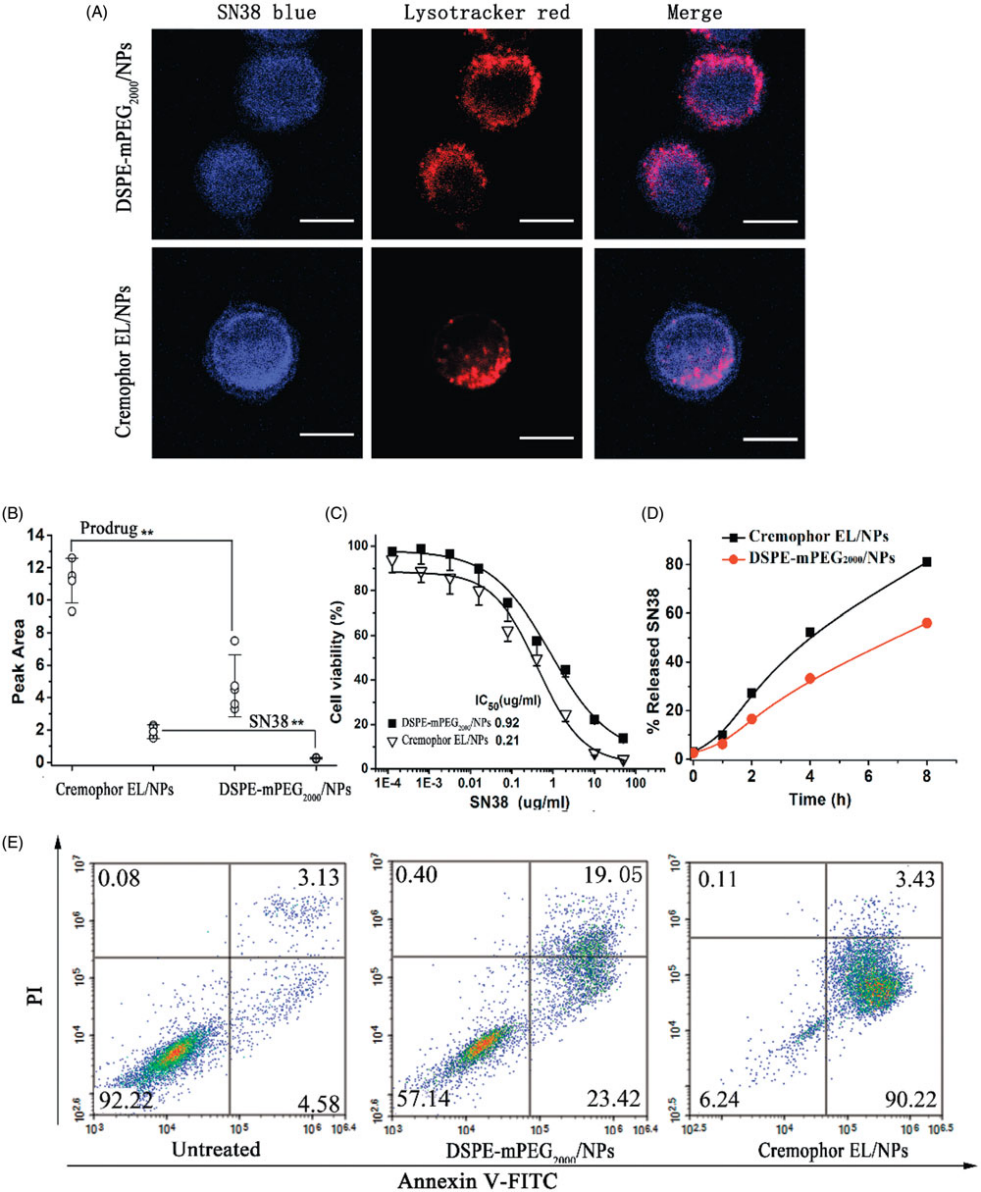
Comparative studies on Cremophor EL/NPs and DSPE-mPEG_2000_/NPs: Confocal laser scanning microscopy images of CT26 cells cultured with NPs at the SN38 equivalent concentration of 10 μg/ml for 2 h (A). HPLC analysis of the released SN38 and prodrug within the CT26 cells cultured with NPs at 10 μg/ml for 4 h [mean ± SD, *n* = 4], **p < .01 (B). Cytotoxicity study against the CT26 determined by the MTT assay (C). SN38 release from Cremophor EL/NPs and DSPE-mPEG_2000_/NPs in the culture medium (D). Apoptotic analysis of CT26 cells by FACS using an Alexa Fluor 488 Annexin V/PI Detection Kit after 48 h of NPs incubation at 5 μg/ml (E).

Such distinct characters of DSPE-mPEG_2000_/NPs and Cremophor EL/NPs were expected to significantly influence the drug delivery performances. We firstly investigated their cellular uptake in CT26 cells using a confocal laser scanning microscopy. CT26 cells were incubated with Cremophor EL/NPs and DSPE-mPEG_2000_/NPs at the SN38 equivalent doses of 10 μg/ml for 2 h at 37 °C before imaging. As shown in Figure 3(A), SN38 signal in blue was primarily localized with lysosomal signal in red, but the extensive blue fluorescence outside the lysosome was also observed. By contrast, Cremophor EL/NPs displayed a stronger intracellular SN38 fluorescence intensity than DSPE-mPEG_2000_/NPs, and SN38 fluorescence was also found in cytomembrane, probably due to the insertion of lipophilic SN38 prodrug into cell membrane (Liu et al., 2017). These results suggested that Cremophor EL-formulated SN38 prodrug was much easier to enter CT26 cells, mainly through a direct transmembrane transportation.

Cellular uptake and intracellular drug release were also investigated using HPLC. As shown in the Figure 3(B), although the intracellular prodrug content after Cremophor EL/NPs incubation was only 2.6-fold higher than that of DSPE-mPEG_2000_/NPs, SN38 released in the cytoplasm reached a high value approaching to 7.4-fold higher than that of DSPE-mPEG_2000_/NPs. This result indicated that lipophilic SN38 prodrug in the Cremophor EL/NPs underwent a faster hydrolysis than that of DSPE-mPEG_2000_/NPs (14.4% versus 5.1%, during 4 h incubation). The distinct intracellular SN38 release rate of NPs might arise from their different pathway of cellular uptake, which was essentially caused by their different kinetic stabilities. DSPE-mPEG_2000_/NPs were mainly endocytosed into acidic lysosomes, where SN38 prodrug was relatively stable even in the presence of elevated reductive GSH (Zheng et al., 2019). For Cremophor EL/NPs with a poor kinetic stability, the lipophilic prodrug of SN38 could be readily released and subsequently insert into cytomembrane. Such transmembrane pathway resulted in the direct entering of the prodrug into the neutral cytoplasm, where SN38 prodrug could rapidly be hydrolyzed to active SN38 in the presence of high concentration of intracellular GSH.

### 3.4. *In vitro* cytotoxicity

I*n vitro* cytotoxicity of DSPE-mPEG_2000_/NPs and Cremophor EL/NPs were evaluated in CT26 cells using the MTT assay. As shown in Figure 3(C), Cremophor EL/NPs showed about 4-fold higher cytotoxicity than that of DSPE-mPEG_2000_/NPs after 48 h incubation, and their IC50 values were comparable to that of free SN38 (0.42 µg/ml) (Zheng et al., 2019). For the better understanding of cytotoxicity, drug release from DSPE-mPEG_2000_/NPs and Cremophor EL/NPs in the culture medium were studied. As shown in Figure 3(D), both versions of NPs showed rapid SN38 release, but Cremophor EL/NPs displayed a relatively faster SN38 release than that of DSPE-mPEG_2000_/NPs (81% versus 56%, within 8 h). This result indicated that the more potent cytotoxicity of Cremophor EL/NPs was ascribed not only to the higher cellular uptake but also to the faster SN38 release in the culture medium.

To further compare their anti-proliferative effect, the fluorescence-activated cell sorting (FACS) analysis was carried out to detect the cells apoptosis after incubation with Cremophor EL/NPs and DSPE-mPEG_2000_/NPs at SN38 equivalent doses of 5 μg/ml for 48 h at 37 °C. As shown in the Figure 3(E), Cremophor EL/NPs showed the higher apoptosis percentage than that of DSPE-mPEG_2000_/NPs (93.65% versus 42.47%). There are two possible explanations accounting for the higher apoptosis ratio for Cremophor EL/NPs. Firstly, Cremophor EL/NPs showed higher cellular uptake and faster drug release in the cytoplasm (Figure 3(A–B)). On the other hand, Cremophor EL/NPs displayed a faster SN38 release than that from DSPE-mPEG_2000_/NPs in the culture medium (Figure 3(D)), which also resulted in higher exposure of active SN38 to cells.

### 3.5. Pharmacokinetics

**Figure 4.**
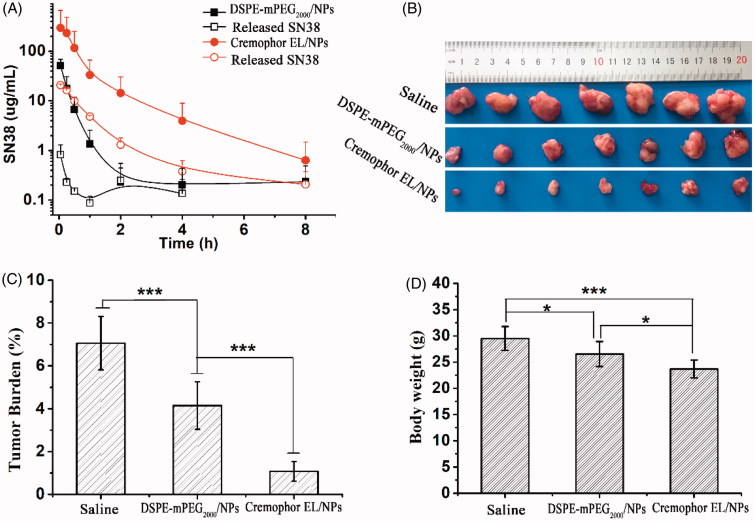
Plasma concentration–time profiles in rat following a single intravenous administration at the SN38 equivalent dose of 5 mg/kg, [mean ± SD, *n* = 4] (A). Tumor picture (B), tumor burden (C) and mice body weight (D) after last treatment against subcutaneous CT26 tumor in BALB/C mice at the equivalent SN38 dose of 5 × 10 mg/kg/2 day, [mean ± SD, *n* = 7], *p <.001, ***p < .001.

Pharmacokinetics was studied in male Sprague–Dawley rats. Cremophor EL/NPs and DSPE-mPEG_2000_/NPs were intravenously injected at the equivalent SN38 dose of 5 mg/kg. As shown in Figure 4(A), the plasma AUC for Cremophor EL/NPs was 13.1 times of that for DSPE-mPEG_2000_/NPs (*p* < .01). This indicated that Cremophor EL/NPs showed a much longer circulating time than that of DSPE-mPEG_2000_/NPs. The relatively rapid clearance of the DSPE-mPEG_2000_/NPs was partly due to the physical blocking of NPs in liver sinusoids as a result of their rod-shape morphology and large particle size, which was generally larger than the diameters (161 nm) of endothelial fenestrae in Sprague–Dawley rats (Wisse et al., 2008). On the other hand, as the prodrug was continuingly dissolved from NPs surface during bloodstream, DSPE-mPEG_2000_ was ready to detach from the surface of NPs. After that, plasma proteins (opsonins) were absorbed on the resulting ‘naked’ NPs, which would be subsequently entrapped in the mononuclear phagocyte system (i.e. liver and spleen) (Rabinow et al., 2007). By contrast, Cremophor EL/NPs, with a very small size (∼15 nm) and distinct drug loading mechanism, was not likely to undergo the clearance mechanism for DSPE-mPEG_2000_/NPs. Therefore, the prolonged circulation time was expected for the Cremophor EL/NPs. These results agreed well with the previous reports, in which DHA-PTX formulated in Cremophor EL exhibited a remarkably higher plasma AUC than that of Taxol both in mice and human (Bradley et al., 2001a; Wolff et al., 2003).

### 3.6. *In vivo* antitumor activity

Subcutaneously grafted CT26 tumor BALB/C male mice (6-8 weeks old) were used to compare the *in vivo* antitumor effects of the Cremophor EL/NPs and DSPE-mPEG_2000_/NPs at the dose of 5 × 10 mg/kg/2 day. As shown in Figure 4(B–D), both DSPE-mPEG_2000_/NPs and Cremophor EL/NPs were able to inhibit the tumor growth as compared with the saline group. However, Cremophor EL/NPs exhibited a remarkably highly *in vivo* antitumor activity over DSPE-mPEG_2000_/NPs (Zheng et al., 2019). This could be attributed to several reasons as following: (a) the longer blood circulation enabled more NPs to accumulate passively into the tumor site. (b) the NPs with an ultra-small size (∼ 15 nm) were expected to penetrate deeply into the tumor parenchyma (Wang et al., 2015). (c) the favorable behavior of cellular uptake enhanced the exposure of active SN38 towards tumor cells. These advantages in the crucial stages of drug deliver resulted in the more potent chemotherapeutic efficacy.

However, Cremophor EL/NPs also exhibited a significant loss of body weight as compared with DSPE-mPEG_2000_/NPs and saline group, indicated that high antitumor activity of Cremophor EL/NPs was also associated with an enhanced systematic toxicity. Such enhanced toxicity could be ascribed to the increased systematic exposure of active SN38 to the normal tissues (i.e. higher AUC of released SN38, Figure 4(A)). On the other hand, the poor kinetic stability of Cremophor EL/NPs also resulted in the rapid leakage of SN38 prodrug and thereafter nonspecific biodistribution, especially upon the infinite blood dilution postintravenous injection (Barenholz, 2012). Therefore, the high toxicity of Cremophor EL/NPs was also attributed, at least in part, to their poor kinetic stability as compared with that of DSPE-mPEG_2000_/NPs. On the other hand, as Cremophor EL-induced hypersensitivity reaction was highly dependent on its concentration in the blood (Gelderblom et al., 2001), the low dose of Cremophor EL could potentially decrease the hypersensitivity reaction caused by Cremophor EL.

## 4. Conclusion

In conclusion, a comparatively investigation on the SN38 prodrug-loaded Cremophor EL/NPs and DSPE-mPEG_2000_/NPs were performed in this study. In comparison with DSPE-mPEG_2000_/NPs, Cremophor EL/NPs with the smaller particle size (∼15 nm) displayed a more efficient delivery performance than that of DSPE-mPEG_2000_/NPs, such as the enhanced cell internalization, higher cytotoxicity and prolonged blood circulation time. These superiorities further resulted in the potent *in vivo* antitumor efficacy in CT26 colorectal cancer xenograft, which suggested that Cremophor EL was more advantageous than DSPE-mPEG_2000_ for the PEGylation of DHP NPs in terms of the improved chemotherapeutic efficacy. As DHPs have been continuingly playing a crucial role in the development of novel nanomedicine for hydrophobic drugs, such comparison is of great importance, especially for design of other more advanced PEGylating agents for efficient delivery of DHPs.
